# Modeling time series by aggregating multiple fuzzy cognitive maps

**DOI:** 10.7717/peerj-cs.726

**Published:** 2021-09-20

**Authors:** Tianming Yu, Qunfeng Gan, Guoliang Feng

**Affiliations:** 1 School of Automation Engineering, Northeast Electric Power University, Jilin, Jilin, China; 2 College of Information and Control Engineering, Jilin Institute of Chemical Technology, Jilin, Jilin, China

**Keywords:** Time series, Fuzzy cognitive maps, Granular computing

## Abstract

**Background:**

The real time series is affected by various combinations of influences, consequently, it has a variety of variation modality. It is hard to reflect the variation characteristic of the time series accurately when simulating time series only by a single model. Most of the existing methods focused on numerical prediction of time series. Also, the forecast uncertainty of time series is resolved by the interval prediction. However, few researches focus on making the model interpretable and easily comprehended by humans.

**Methods:**

To overcome this limitation, a new prediction modelling methodology based on fuzzy cognitive maps is proposed. The bootstrap method is adopted to select multiple sub-sequences at first. As a result, the variation modality are contained in these sub-sequences. Then, the fuzzy cognitive maps are constructed in terms of these sub-sequences, respectively. Furthermore, these fuzzy cognitive maps models are merged by means of granular computing. The established model not only performs well in numerical and interval predictions but also has better interpretability.

**Results:**

Experimental studies involving both synthetic and real-life datasets demonstrate the usefulness and satisfactory efficiency of the proposed approach.

## Introduction

Time series data modeling and prediction has always been a classic research topic, which can predict the future by analyzing, understanding and modeling historical data. Time series prediction has been widely used in many fields, such as economy, industry, climate prediction (Zeng & Khondker, 2016), medical (Ma et al., 2018; Zheng et al., 2018; Nishii et al., 2016), and so on. Its significance lies in its ability to provide the basis for decision-making in these fields effectively (Weigend & Gerschenfeld, 1994). Linear system theory, stochastic process theory (Yin et al., 2019; Ren, Zhang & Zhang, 2019; Zhang & Wang, 2021), black box theory and other methods have been used by researchers to develop many classic time series numerical models, such as autoregressive integrated moving average (ARIMA) (Lee & Tong, 2011), neural network models (Zhang, 2003; Khashei, Bijari & Ardali, 2012), support vector machines (Cao & Tay, 2003; Chaâbane, 2014), Bayesian networks (Guo, Liu & Sun, 2016) and other models. These models have been widely used in various fields and show good performance in numerical prediction. However, these numerical models are difficult to be understood due to their low interpretability. In order to make the time series model interpretable and maintain the robustness of uncertainty, Fuzzy Set Theory provides a feasible choice. Fuzzification is introduced into modeling the time series, and then the fuzzy time series model is constructed (Song & Chissom, 1993). The main steps of the fuzzy time series model are as follows: the division of the interval of the domain of definition, the language description of the interval, the fuzzification of the time series, the establishment of the fuzzy logic relationship among variables of fuzzy time series, the calculation of predicted value and defuzzification (Singh, 2017; Bose & Mali, 2019). In addition, in some fields, only an approximation value is enough, so that it is not necessary to know the accurate value of the time series. For example, for daily temperature forecasts, the minimum and maximum values of temperature are more instructive. In the stock market, investors are more interested in the range of decline and rise in the future. In these fields, people prefer to model and forecast time series at the level of symbol or information granularity, rather than just predict the accurate value. In order to construct a higher-level representation method of the time series model, a time series modeling method based on granular computing is further proposed by researchers (Lu, Chen & Pedrycz, 2015; Froelich & Pedrycz, 2017). Granular computing (Bargiela & Pedrycz, 2008) is a human-centered information processing framework platform, which uses “reasonable information granulation principle” (Pedrycz, 2011; Pedrycz & Homenda, 2013) to divide complex and abstract information into simple and understandable information granules according to certain rules. This method does not excessively pursue the accurate value of the model, but mediates the “precision” and “interpretability” of the model so that the dynamic behavior of time series is easier to be understood.

As a soft computing tool, Fuzzy Cognitive Maps (FCMs) realize the reasoning process based on knowledge representation and can capture the behavior of a given dynamic system, so it is used to describe and model complex systems (Papageorgiou & Salmeron, 2012; Felix et al., 2019). FCMs are composed of concept nodes and directed weights. Nodes represent the main behavior characteristics of the system, and weights reflect the causal relationship between concept nodes. Based on the knowledge reasoning ability of FCMs, Stach, Kurgan & Pedrycz (2008) used real coded genetic algorithm to learn fuzzy cognitive map and predict time series at numerical and linguistic levels. Lu, Yang & Liu (2014) employed the prior states of the nodes to forecast the current states to improve the performance of FCMs models, which is termed as high-order fuzzy cognitive maps. Pedrycz, Jastrzebska & Homenda (2016) designed a scheme that uses information granules to describe numerical time series. In the space of amplitude and amplitude change of time series, the fuzzy c-means clustering algorithm is used to cluster the data, and each cluster center is used as a node of FCMs to model and predict the time series. Salmeron & Froelich (2016) used strategy of dynamic optimization to update the weight of FCMs dynamically to improve the prediction accuracy of time series model. This method dynamically adjusts the weights of FCMs according to the local characteristics of time series. Froelich & Pedrycz (2017) used the principle of reasonable granularity and the principle of fuzzy c-means clustering to approximate the granularity of time series. In this way, a compromise between particularity and universality is de-signed when generating approximate values of time series. In addition, the time series can be more easily understood by human beings after granulating the obtained particles and assigning language items, which is a character that the method of directly analyzing the original data does not have. Feng et al. (2021) presented a rapid and robust FCMs learning method with maximum entropy, and developed a long-term time series prediction FCMs model based on this learning method (Feng et al., 2021).

When modeling time series, the existing methods usually model the entire time series as a whole, that is, assuming that the time series has only one pattern of variation. However, the change patterns of time series in different time periods and intervals are not the same. In addition, the main time series may also be derived from multiple objects. Therefore, it is difficult to mine all the change characteristics of time series by modeling the whole time series directly. If each change mode of time series can be modeled, the knowledge representation of time series will be more comprehensive. In this paper, a large number of short sequences are selected in a given time series by using a random strategy with putting back in order to contain various variation modes in the time series. Then, Fuzzy Cognitive Maps is used to model each subsequence. The output of each model is fused by the method of granulation, and finally, the output of the time series is obtained. The proposed method can not only obtain the numerical prediction value of the time series but also obtain the prediction interval. Furthermore, it has semantic descriptions, which makes it easier for people to understand the change characteristics of time series.

## Materials & Methods

### Fuzzy cognitive maps

**Figure 1:**
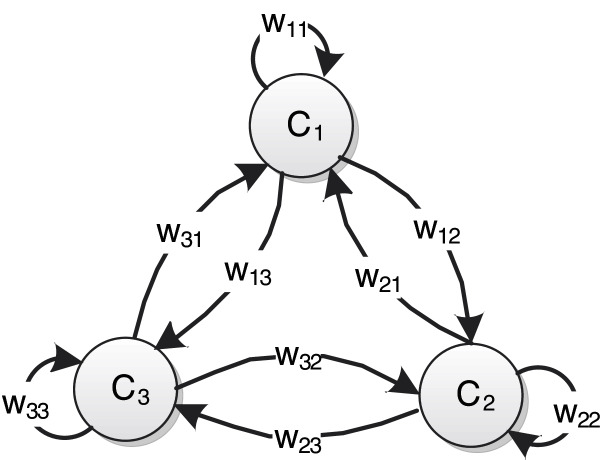
An example of FCMs consisting of three nodes.

The topological structure of FCMs is a weighted directed graph with feedback loops, which describe the characteristics of the physical system through nodes and weight edges. As shown in Fig. 1, a typical FCMs is composed of weight edges between conceptual nodes and nodes. These nodes represent the main variables in the system that to be studied, and the weighted edges represent the degree of mutual influence among nodes.

The semantics of standard FCMs can be represented by a 4-tuple $\left( {\bi {C},\bi {W},\bi {A},{\it f}} \right)$ (Felix et al., 2019). Suppose that FCMs has n nodes, ${\bi {C}} = \left\{ {{C_1},{C_2}, \cdots ,{C_n}} \right\}$ is a set of *n* conceptual nodes, and $\bi {W}$ is the weight matrix of *n* × *n* dimensional:

(1) $${\bi {W}} = \left[ {\matrix{ {w_{11}}  {w_{12}}  {\cdots}  {w_{1n}} \cr {w_{21}}  {w_{22}}  {\cdots}  {w_{2n}} \cr {\vdots}  {\vdots}  {}  {\vdots} \cr {w_{n1}}  {w_{n2}}  {\cdots}  {w_{nn}} \cr } } \right]$$

where plus, minus and value size of ${{\rm w}_{{\rm ij}}} \in \left[ { - 1,1} \right]$ respectively reflect the direction and degree of causality between nodes. ${w_{ij}} > 0$ indicates that ${C_j}$ will increase with the increase of ${C_i}$, and the degree of influence is ${w_{ij}}$, and vice versa. ${w_{ij}} < 0$ denotes that ${C_j}$ will decrease with the increase of ${C_i}$, and the degree of influence is $\left| {{w_{ij}}} \right|$, and vice versa. ${w_{ij}} = 0$ means that there is no causal relationship between ${C_i}$ and ${C_j}$. ${\bi {A}}:{C_i} \to {A_i}\left( t \right)$, ${A_i}\left( t \right)$ is the state value of the node ${C_i}$ at the moment $t$, the value changes dynamically. $f$ is the activation function, which is a nonlinear monotone increasing function, it integrates the input state values of all nodes associated with the target node into the definitional domain of the activation function. The state value of the node ${C_j}$ at the time $t + 1$ can be obtained by the following formula:

(2) $${A_j}\left( {t + 1} \right) = f\left( {\mathop \sum \limits_{i = 1}^n {A_i}\left( t \right){w_{ij}}} \right)$$

The Sigmoid function is a common activation function:

(3) $$f\left( x \right) = \displaystyle{1 \over {1 + {e^{ - \lambda x}}}}{\rm \; }$$

This activation function limits the state value of the node to the interval [0, 1], where ${\rm \lambda } > 0$ determines the steepness of the activation function.

### Granular computing

Granular computing was first proposed by Zadeh (1997, 2008), and the general definition of fuzzy information granule was given as follows:

(4) $$g = \left( {x{\rm \; }is{\rm \; }G} \right){\rm \; }is{\rm \; }\mu$$

where $x$ denotes a variable on the discourse universe $U$, $G$ is a fuzzy subset of $U$, $\mu$ is the probability that $x$ belongs to the fuzzy set $G$. $g$ is an information granule, its lexeme is “the probability level of $x$ belongs to the fuzzy set $G$ is $\mu$”. Granular computing is embodied in the process of data abstraction and information knowledge representation, different levels of abstraction can be represented by different sizes of information granules.

Bargiela & Pedrycz (2008) and Pedrycz (2018) further developed the interpretability research of granular computing on this basis and regarded information granular as an information processing process of “human-centered”, which was used for knowledge representation of actual models and decision-making process.

Granular computing follows the principle of justifiable granularity, that is, reasonable information granulation should represent the data as much as possible, on the other hand, data should be abstracted as much as possible and expressed semantically. In short, the principle of granular computing is manifested in two aspects: coverage and specificity (Pedrycz & Vukovich, 2001). The coverage denotes how much data is covered by the constructed information granules, it requires data to be covered by information granular as possible. The specificity means that the generated information granular should have clear semantic meaning, the constructed information granular is required to be as small as possible, so that the semantic is clearer. It can be seen that the requirements of the two are contradictory.

Suppose there is a data set ${\bi {Y}} = \left\{ {{y_1},{y_2}, \cdots ,{y_p}} \right\}$. The coverage of the information granular is quantified by determining the number of elements in the data set covered by the information granule. Assume $i \in \left[ {1,p} \right]$, the coverage cov of information granular is defined as follows:

(5) $$cov = card\left\{ {{y_i}|{y_i} \in g} \right\}$$

$card\left( \cdot \right)$ represents the cardinality, that is, how many elements of the data set $\bi {Y}$, belong to the information granule $g$. The specificity is quantified by the width of information granule $g$, and the specificity sp is defined as follows:

(6) $$sp = {\rm exp}\left( { - \alpha \left| {U - L} \right|} \right)$$

where ${\rm U}$ and $L$ respectively represent the upper and lower bounds of the amplitude of the data covered by the information granule. The smaller the distance U–L between the upper and lower bounds, the more specific the information granule. $\alpha > 0$, the level of information granularity provides flexibility for the constructed information granular, and its value affects the specificity of the information granular.

The key to information granulation is to construct a function to make the coverage and specificity of information granular better. However, increasing the coverage of information granular leads to the decrease of specificity, and vice versa. To balance these two characteristics, the problem of constructing information granular is transformed into the following optimization problem:

(7) $$\arg \mathop {\max }\limits_{U,L} {\rm \; }J = cov \times sp$$

## The Proposed Method

When fuzzy cognitive maps is used to model time series, the following four steps are generally followed: fuzzification of time series, learning of fuzzy cognitive maps, modeling and prediction, and defuzzification of predicted value (Stach, Kurgan & Pedrycz, 2008). This section will describe the whole modeling process in detail

### Fuzzification of time series

For a time series ${\bi {X}} = \left\{ {{x_1},{x_2}, \cdots ,{x_m}} \right\}$, $m$ is the number of sample data contained in the time series. A triangular fuzzy set is used to fuzzify the time series $\bi {X}$. The membership function formula of the triangular fuzzy set is as follows:

(8) $$\mu \left( {x;a,b,c} \right) = \left\{ {\matrix{ {\matrix{ 0 & {x \le a} \cr } } \cr {\matrix{ {\displaystyle{{x - a} \over {b - a}}} & {a \le x \le b} \cr } } \cr {\matrix{ {\displaystyle{{c - x} \over {c - b}}} & {b \le x \le c} \cr } } \cr {\matrix{ 0 & {x \ge c} \cr } } \cr } } \right.$$

where parameters $a$ and $c$ correspond to the left and right vertices of the triangle, $b$ is the middle vertex of the triangle, and $a \le b \le c$.

Suppose that the definitional domain of time series $\bi X$ is divided into continuous and disjoint intervals by $n$ points $\left\{ {{P_1},{P_2}, \cdots ,{P_n}} \right\}$, and each division point is a fuzzy linguistic variable. Then the membership degree of sample data ${x_i}$ to these fuzzy sets is expressed as $\left\{ {{\mu _1}\left( {{x_i}} \right),{\mu _2}\left( {{x_i}} \right), \cdots {\mu _n}\left( {{x_i}} \right)} \right\}$.

After the time series ${\bi {X}} = \left\{ {{x_1},{x_2}, \cdots ,{x_m}} \right\}$ is fuzzified by triangular fuzzy set, the matrix $\bi {U}$ formed by the fuzzy time series can be obtained as follows:

(9) $${\bi {U}} = \left[ {\matrix{ {u_{11}}  {u_{12}}  {\cdots}  {u_{1n}} \cr {u_{21}}  {u_{22}}  {\cdots}  {u_{2n}} \cr {\vdots}  {\vdots}  {}  {\vdots} \cr {u_{m1}}  {u_{m2}}  {\cdots}  {u_{mn}} \cr } } \right]$$

where $m$ is the number of samples of time series $\bi {X}$ and $n$ is the number of the divided fuzzy sets. The elements in each row of $\bi {U}$ are the fuzzy membership values of the corresponding time series samples, and the sum of elements in each row is 1. The vector of triangular fuzzy linguistic variables is as follows:

(10) $${\bi {P}} = \left[ {{P_1},{P_2}, \cdots ,{P_n}} \right]$$

### Modeling with fuzzy cognitive maps

After the fuzzy time series is determined, it can be used for learning FCMs. As a symbolic reasoning mechanism, the reasoning Eq. (2) of FCMs can be written as follows:

(11) $${\bi{A}}_j\left( {t + 1} \right) = f\left( {{\bi{A}}\left( t \right){{\bi{W}}_j}} \right)$$

where ${\bi{A}}\left( t \right) = \left[ {{\bi{A}_1}\left( t \right),{\bi{A}_2}\left( t \right), \cdots {\bi{A}_n}\left( t \right)} \right]$ are the state values of all nodes of FCMs at time $t$, ${\bi{A}}_j\left( {t + 1} \right)$ is the state value of the *j*-th node of FCMs at the time $t + 1$, and ${\bi{W}}_j = {\left[ {{w_{1j}}{\rm \; }{w_{2j}}{\rm \; } \cdots {\rm \; }{w_{nj}}} \right]^T}$ is the *j*-th column of the weight matrix $\bi{W}$. As activation function, a Sigmoid function has a unique inverse function, so after the Eq. (11) was inversely transformed, it can be expressed as:

(12) $${f^{ - 1}}\left( {{\bi{A}_j}\left( {t + 1} \right)} \right) = {\bi{A}}\left( t \right){{\bi{W}}_j}$$

The above equation is linear. Where ${f^{ - 1}}\left( {\rm y} \right) = - \textstyle{1 \over \lambda }\ln \textstyle{{1 - y} \over y}$ is the inverse function of the Sigmoid function.

After the fuzzy time series matrix $\bi {U}$ is obtained, it is used for learning the weight $\bi {W}$ of FCMs. Let ${\bi{Z}} = {\left[ {{{\bi{U}}_1},{{\bi{U}}_2}, \cdots ,{{\bi{U}}_{m - 1}}} \right]^\bi{T}}$, where ${\bi{U}}_i = \left[ {{u_{i1}}{\rm \; }{u_{i2}}{\rm \; } \cdots {\rm \; }{u_{in}}} \right]$, ${\bi{Y}}_j = {f^{ - 1}}\left( {{{\left[ {{u_{2j}}{\rm \; }{u_{3j}}{\rm \; } \cdots {\rm \; }{u_{mj}}} \right]}^\bi{T}}} \right)$, put $\bi {Z}$ and $\bi Y_j$ into Eq. (12):

(13) $${\bi{Y}}_j = {\bi{Z}\bi{W}}_j$$

$\bi{Z}$ and ${\bi{Y}}_j$ can be regarded as independent variables and dependent variables of historical data respectively. Therefore, the learning problem of FCMs weight can be transformed into the solution of the least square problem. The following objective function is constructed to solve $\bi{W}_j$:

(14) $${\rm arg\; \; }\mathop {\min }\limits_{{{W}_j}} \|{{Z}{W}}_j} - {{Y}}_j\|_2$$

$${\rm s}.{\rm t}.{\rm \; }\|{W_j}\|{_\infty } \le 1$$

$\|{\bi {Z}\bi{W}}_j - {\bi{Y}}_j\|_2$ in the objective function is used to minimize the least square error between the actual value and the predicted value, to obtain a reasonable approximate solution of ${\bi{W}_j}$. The bound term $\|{\bi{W}}_j\|_\infty \le 1$ ensures that the value of each element of the weight matrix is in the interval [−1, 1], where the infinite norm $\|{\bi{W}}_j\|_\infty = \max \left\{ {\left| {{w_{1j}}} \right|,\left| {{w_{2j}}} \right|, \cdots ,\left| {{w_{nj}}} \right|} \right\}$. The above formula is a standard convex optimization problem with linear constraints. At present, there are many convex optimization techniques to solve the problem, such as the commonly used interior point method and primal-dual method (Boyd & Vandenberghe, 2004).

The prediction of time series is based on historical data to infer future data. In this paper, the reasoning mechanism of FCMs is used to predict time series. The state vector of FCMs at the time $t$ is given as ${\bi{U}}_t = \left[ {{u_{t1}}{\rm \; }{u_{t2}}{\rm \; } \cdots {\rm \; }{u_{tn}}} \right]$, once the weight matrix $\bi{W}$ is determined, then the predicted value ${\hat {\bi{U}}_{t + 1}}$ of the state vector at time $t + 1{\rm \; }$ can be calculated by the following formula:

(15) $${\hat {\bi{U}}_{t + 1}} = f\left( {{\bi{U}_t}\bi{W}} \right)$$

Through the above formula, the state vector at the next moment of FCMs can be obtained, so the prediction matrix $\hat {\bi{U}}$ of the state vector can be obtained. In order to obtain the final value of time series prediction, it is necessary to defuzzify the state vector, the formula is as follows:

(16) $${\hat {\bi{x}}_{t + 1}} = \displaystyle{{\mathop \sum \nolimits_{j = 1}^n {\hat {\bi{u}}_{\left( {t + 1} \right)j}}{P_j}} \over {\mathop \sum \nolimits_{j = 1}^n {\hat {\bi{u}}}_{\left( {t + 1} \right)j}}}}$$

where $n$ is the number of nodes of FCMs, *P*_*j*_ is the triangular fuzzy linguistic value of the *j*-th node of FCMs, as shown in Eq. (10).

### Aggregation of FCMs models

The data in time series change over time and come from various sources, that is, time series usually contain multiple modal features. To understand the characteristics of time series more deeply, it is necessary to build a model that can mine and use these modal features for modeling. Moreover, direct modeling of large time series will lead the model to be too complicated to understand. Therefore, the multi-modal modeling method is used to analyze the time series, to mine the change characteristics of the time series subsystem, and finally to represent the change characteristics of the whole time series.

**Figure 2:**
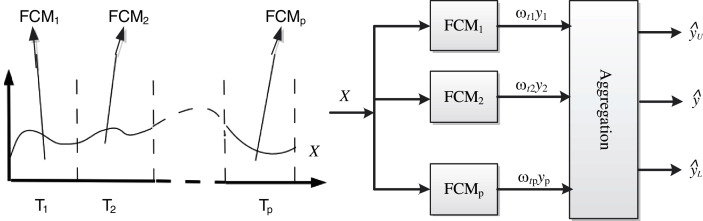
Modeling framework.

Figure 2 shows the framework of the FCMs multimodal modeling system, Bootstrap is used to randomly extract $p$ subsets from the time series $\bi{X}$, and each subset trains an FCMs sub-model. Then the outputs of each sub-model are fused by the information granulation method to obtain the final output value and its upper and lower limit value.

The specific steps of the multi-mode FCMs model are as follows:

(1) For the time series $\bi X$ of length $m$, the triangular fuzzy set with the number of fuzzy sets $n$ is used for fuzzification, and the fuzzy time series $\bi{U}$ is obtained.
(2) The bootstrap method is used to randomly select $p$ subsets from fuzzy time series $\bi {U}$ of length *m*. It should be noted that the subsequences are not necessarily adjacent in order. In this study, the starting point of each subsequence is randomly selected, and the lengths of the subsequences are the same. Set the length of each subsequence to $k$, then the *i*-th ($i = 1,2, \cdots ,p$) subsequence can be represented as follows:

(17) $${{\bar {\bi U}^i}} = \left[ {\matrix{ \bar u_{11}^i  \bar u_{12}^i  {\cdots}  \bar u_{1n}^i \cr \bar u_{21}^i  \bar u_{22}^i  {\cdots}  \bar u_{2n}^i \cr {\vdots}  {\vdots}  {}  {\vdots} \cr \bar u_{k1}^i  \bar u_{k2}^i  {\cdots}  \bar u_{kn}^i \cr } } \right]$$

(3) For each subsequence ${{\bar {\bi U}^i}}$, the method described in the previous section is used for FCMs modeling, and $p$ FCMs models are obtained.
(4) How to fuse the output of the sub-model is the key to realize the multimodal model. Suppose that the weights of $p$ models are ${\omega _1},{\omega _2}, \cdots ,{\omega _p}$ respectively, the output and the weights of a model form a set of weighted data set ${\bi{Y}} = \left\{ {\left( {{y_1},{\omega _1}} \right),\left( {{y_2},{\omega _2}} \right), \cdots ,\left( {{y_p},{\omega _p}} \right)} \right\}$, where ${\omega _i} \in \left[ {0,{\rm \; }1} \right]$ is the weight of data ${y_i}$($i = 1,2, \cdots ,p$). Granulating the information of data set $\bi{Y}$, and the optimization objective function is constructed as follows:

(18) $$\arg \mathop {\max }\limits_{U,L} {\rm \; }J = cov \times sp \times flag$$

where the constructed coverage function is $cov = \sum {\omega _i},{\rm \; }y\left( i \right) \in \left[ {{{\hat y}_L},{{\hat y}_U}} \right],i \in \left[ {1,p} \right]$, the special function is $sp = {\rm exp}\left( { - \alpha \left| {{{\hat y}_L} - {{\hat y}_U}} \right|} \right)$, ${\hat y_L}$ and ${\hat y_U}$ respectively represent the upper and lower bounds of the data amplitude covered by information granule. When it is used for time series prediction, the $flag$ represents whether the actual value at the previous moment is within the upper and lower bounds:

$$flag = \left\{ {\matrix{ {\; \; 1,\; \; \; y \in \left[ {{{\hat y}_L},{{\hat y}_U}} \right]} \cr {0,\; \; \; \; \; \; \; \; \; \; \; other\; } \cr } } \right.$$

The purpose of constructing the objective function is to make the weight of the data covered by the constructed information granule as large as possible, and the interval of the information granule as small as possible. When making interval prediction for time series, it is always hoped that the obtained interval value should be as small as possible, but at the same time, the actual value should be kept within the prediction interval. The physical meaning of the interval obtained by (18) is the upper and lower boundary of the prediction interval.

(5) Suppose that there are q data in the interval of the information granule obtained in the previous step, the weights of these data obtained by recalculation are ${\lambda _1},{\lambda _2}, \cdots ,{\lambda _q}$, and the calculation formula of the final prediction value is:

(19) $$\hat y = \mathop \sum \limits_{i = 1}^q {\lambda _i}{y_i}{\rm \; }$$

where ${y_i} \in \left[ {{{\hat y}_L},{{\hat y}_U}} \right]$, ${\hat y_L}$ and ${\hat y_U}$ represent the upper and lower bounds of the prediction interval respectively.

Root mean square error (RMSE) is used to evaluate the prediction performance of the model:

(20) $$RMSE = \sqrt {\displaystyle{1 \over N}\mathop \sum \limits_{t = 1}^N x\left( t \right) - y{{\left( t \right)}^2}}$$

where $x\left( t \right)$ denotes the actual value at time $t$, and $y\left( t \right)$ represents the simulated value at time $t$. Obviously, the smaller the RMSE value is, the better the prediction performance of the model is. $N$ is the number of data.

In order to obtain the final output of the multimodal model, it is necessary to determine the weight of each sub-model. The weighting strategy uses the following three methods:

(1) Average weight

Use the arithmetic average of each sub-model output as the output of the multi-modal system, that is, the weight of $p$ sub-models is 1/*p*. The weight formula ${\omega _i}$ of the $i$-th sub-model is as follows:

(21) $${\omega _i} = \displaystyle{1 \over p}{\rm \; }$$

(2) Model weight

The RMSE of each model is calculated. The smaller the RMSE is, the greater the weight of the corresponding sub-model is. Take the reciprocal of the RMSE of each sub-model and normalize it. Take the reciprocal of each RMSE and normalize it.

(22) $${\omega _i} = {\rm \; }\displaystyle{{1/RMS{E_i}} \over {\mathop \sum \nolimits_{i = 1}^p 1/RMS{E_i}{\rm \; }}}$$

(3) Dynamic weight

The absolute difference between the predicted value and the actual value at the last moment is used to determine the weight of the model. In this way, the weight of each sub-model changes in real-time. The weight formula ${{\rm \omega }_{\rm i}}\left( {\rm t} \right)$ of the *i*-th sub-model is as follows:

(23) $${\omega _i}\left( t \right) = {\rm \; }\displaystyle{{1/\left| {{x_i}\left( {t - 1} \right) - {y_i}\left( {t - 1} \right)} \right|} \over {\mathop \sum \nolimits_{i = 1}^p 1/\left| {{x_i}\left( {t - 1} \right) - {y_i}\left( {t - 1} \right)} \right|{\rm \; }}}$$

After the weight of the sub-model is determined, the output of the multimodal model is calculated as follows:

(24) $$\hat y = \mathop \sum \limits_{i = 1}^p {\omega _i}{y_i}{\rm \; }$$

## Results

RMSE is used to evaluate the numerical prediction performance of the constructed model, and the formula is shown in (20). PICP (Prediction interval coverage probability) and PINAW (Prediction interval normalized average width) are used to evaluate interval prediction performance (Khosravi, Nahavandi & Creighton, 2013; Quan, Srinivasan & Khosravi, 2013), and the formula is as follows:

(25) $$PICP = \displaystyle{1 \over N}\mathop \sum \limits_{t = 1}^N {\delta _t}$$

(26) $$PINAW = \displaystyle{1 \over {NR}}\mathop \sum \limits_{t = 1}^N \left( {{{\hat y}_U} - {{\hat y}_L}} \right)$$

where $N$ is the number of prediction samples, ${\delta _t}$ is a Boolean value: ${\delta _t} = \left\{ {\matrix{ {1,\; \; \; x \in \left[ {{{\hat y}_L},{{\hat y}_U}} \right]} \cr {0,\; \; \; x\ \notin \left[ {{{\hat y}_L},{{\hat y}_U}} \right]} \cr } } \right.$. $x$ is the actual value, ${\hat y_L}$ and ${\hat y_U}$ are the lower and upper bounds of the prediction interval respectively, $R$ is the range of amplitude changes of time series data. Based on these two evaluation indexes, CWC (coverage width based criterion) is constructed:

(27) $$CWC = PINAW\left( {1 + PICP \times {e^{ - PICP}}} \right)$$

Among these evaluation indexes, RMSE is used to evaluate the error between the predicted value and the true value. The smaller the RMSE is, the better the performance of the prediction model is. PICP and PINAW are used to evaluate the indicators of the prediction interval. The larger the PICP, the higher the probability that the prediction interval contains the actual sample values. The smaller the PINAW is, the smaller the prediction interval is. An excellent prediction model should have the characteristics of high PICP and small PINAW, CWC, RMSE.

**Figure 3:**
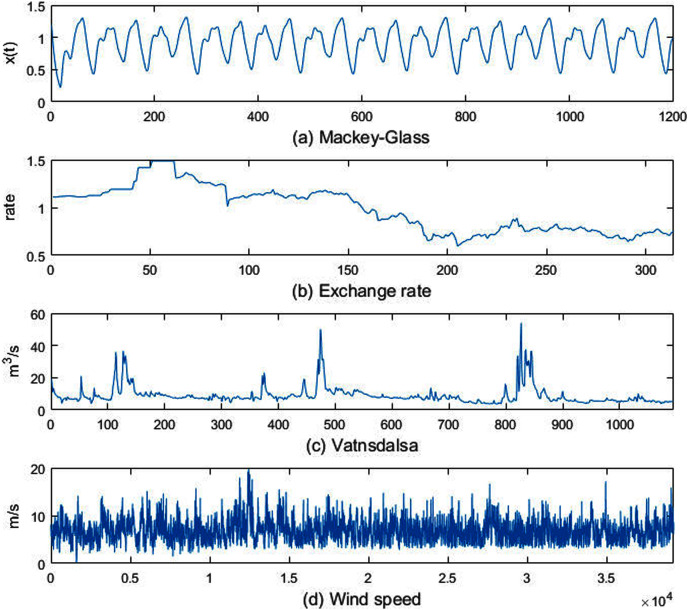
Experimental datasets.

In order to demonstrate the working process of the method proposed in this paper and verify its effectiveness, it experiments with artificial and actual data sets. The data sets include both small and large time series:

(1) Mackey-Glass (MG). Mg is a chaotic, aperiodic, non-convergent sequence, which is obtained by the following delay differential equation:

(28) $$\displaystyle{{d{x_t}} \over {dt}} = \displaystyle{{0.2{x_{t - \tau }}} \over {1 + x_{t - \tau }^{10}}} - 0.1{x_t}$$

let $x_{0}=1.2$, $\tau =17$, when $t \lt 0$, ${x_t} = 0$, Substituting these parameters into the above formula, and the resulting sequence contains 1,201 samples, the sequence is shown in Fig. 3A.

(2) Exchange rate. A time series of the monthly exchange rate between the Australian dollar and the US dollar. The data set contains 314 samples of monthly exchange rates from July 1969 to August 1995, as shown in Fig. 3B.
(3) Vatnsdalsa. The average daily flow (m^3^/s) of the Vatnsdalsa River from January 1, 1972 to December 31, 1974, there are 1,095 samples, as shown in Fig. 3C.
(4) Wind speed. The time interval of the data set is 10 min, and there are 39,195 samples, as shown in Fig. 3D.

In the experiment, the first 80% of the above time series data set samples were used as the training set and the last 20% as the test set. The parameters of the model are shown in Table 1. In order to reduce the complexity of the model, the number of nodes in each sub-FCMs model is $n =3$, the number of samples in each subsequence $k =5$, the number of sub-sequences randomly selected by the bootstrap method is $p =100$, and the granularity level $\alpha =1$.

**Table 1:** Model parameter setting.

Number	Parameters	Values
1	λ	5
2	Submodel number $p$	100
3	Node number $n$	3
4	Subsequence length $k$	5
5	Granularity level $\propto$	1

The experimental platform is a laptop computer with a CPU of 2.3 GHz and a memory of 4G. The software is Matlab R2018a. In the experiment, the starting point of each sampled data is different, and the change of each sub-sequence is also different. Therefore, the FCMs models trained by these subsequences are also different. The purpose of using a large number of random selection methods is to include the changes of time series data as much as possible.

According to the semantics given by the fuzzy set of the data domain, the three nodes of FCMs are defined as low amplitude, medium amplitude, and high amplitude. When the triangular fuzzy set is used for the fuzzification of the time series, the minimum, median and maximum values of the time series are selected as the linguistic variables of the fuzzy set. Table 2 shows the specific values corresponding to the node semantics in the FCMs model corresponding to each data set. Taking the MG sequence as an example, the value corresponding to the semantic “low amplitude” is 0.2192, the value corresponding to “medium amplitude” is 0.7665, and the value corresponding to “high amplitude” is 1.3117. When the semantic description of the language variable is given, the predicted value output by the model can be classified into corresponding semantics according to the membership degree.

**Table 2:** The semantics of FCMs nodes.

Data	Low	Medium	High
MG	0.2192	0.7665	1.3137
Exchange rate	0.5980	1.0430	1.4880
Vatnsdalsa	3.6700	28.8350	54.0000
Wind speed	0.0900	9.9400	19.7900

## Discussion

**Figure 4:**
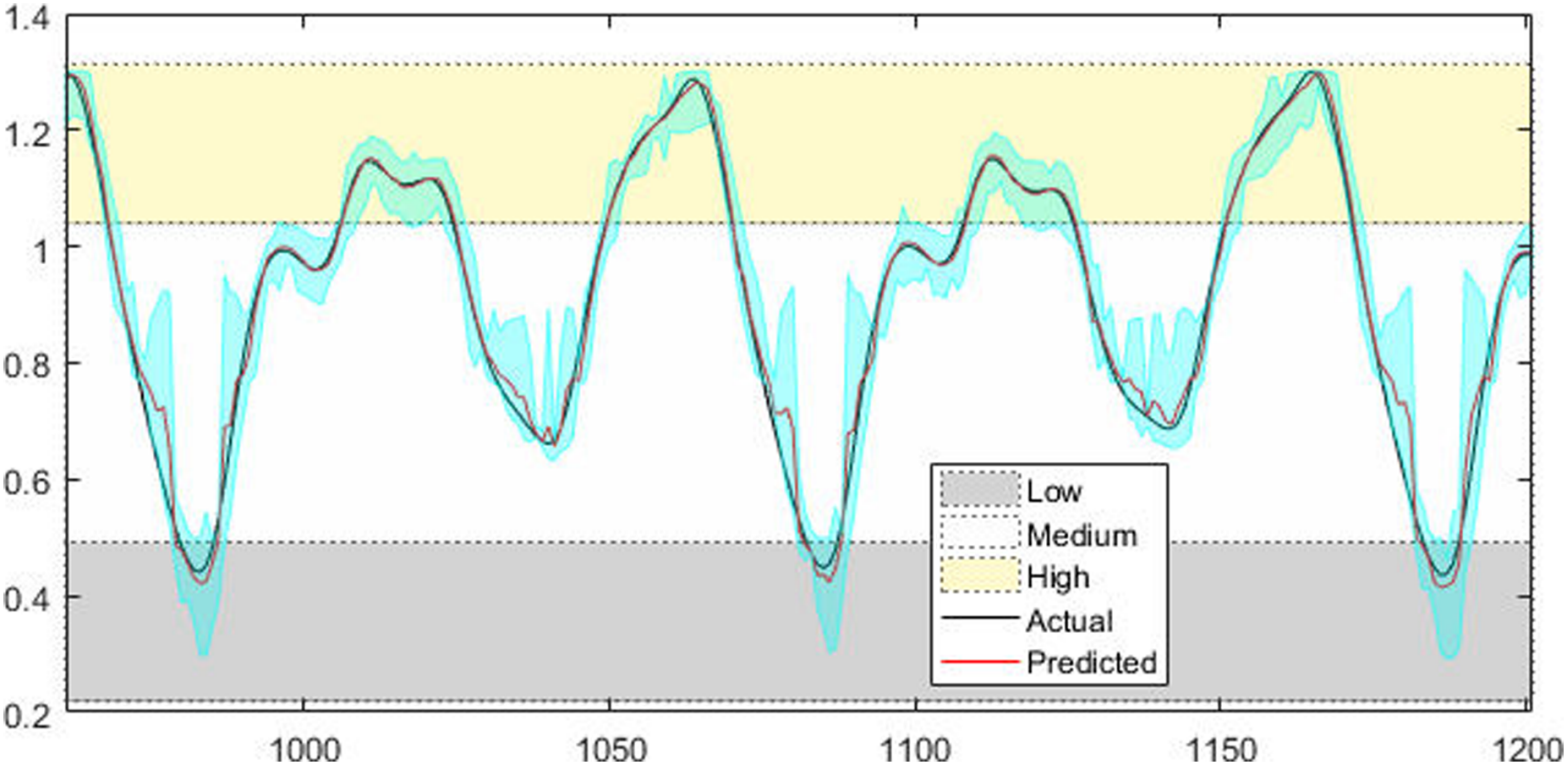
The prediction results of MG time series.

**Figure 5:**
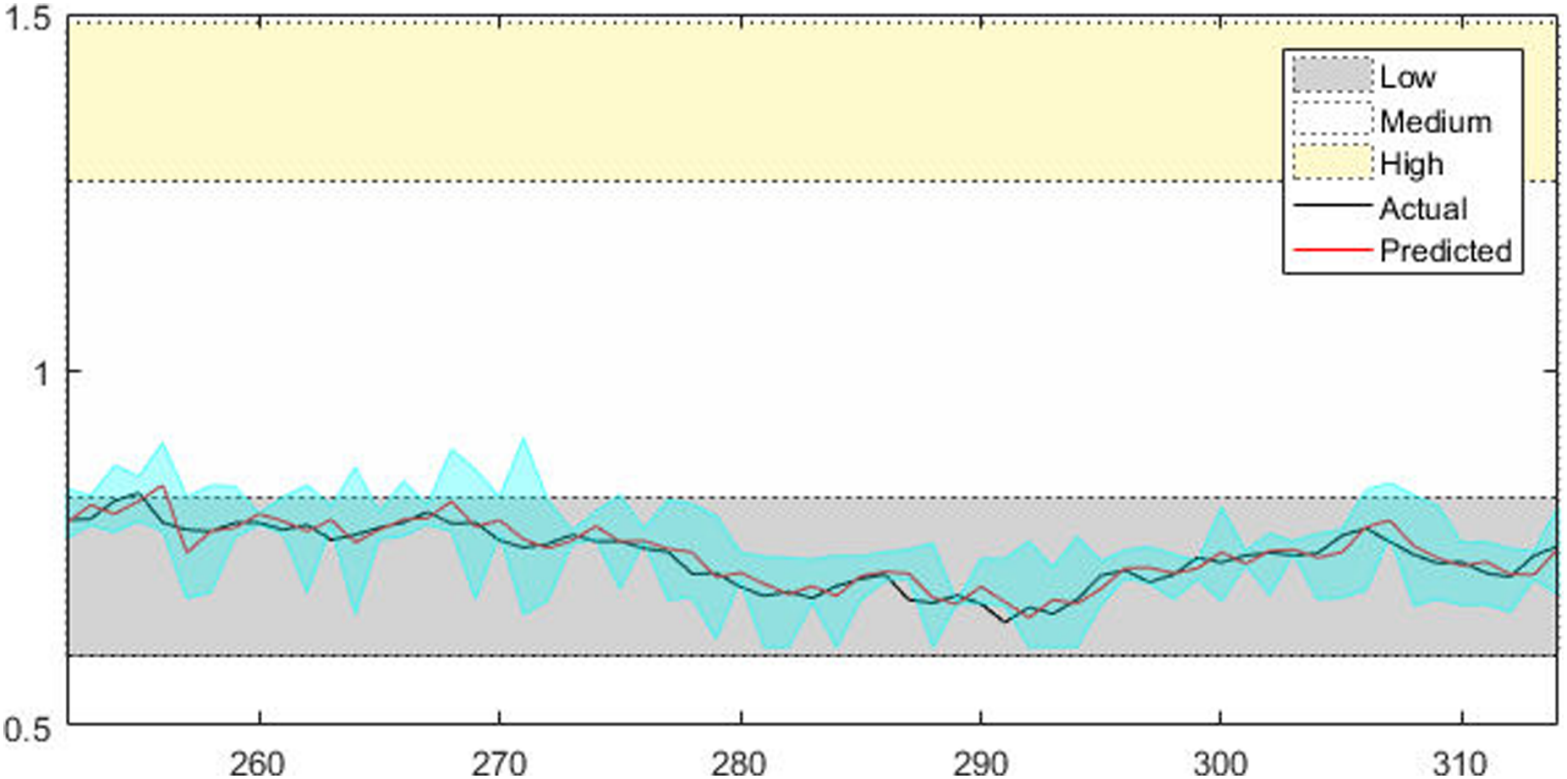
The prediction results of AUD/USD time series.

**Figure 6:**
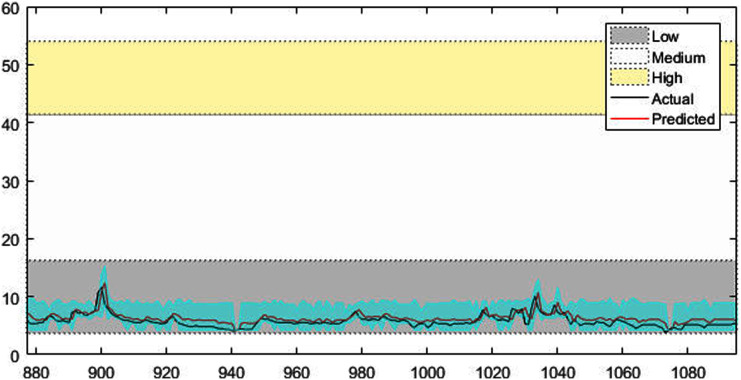
The prediction results of Vatnsdalsa time series.

**Figure 7:**
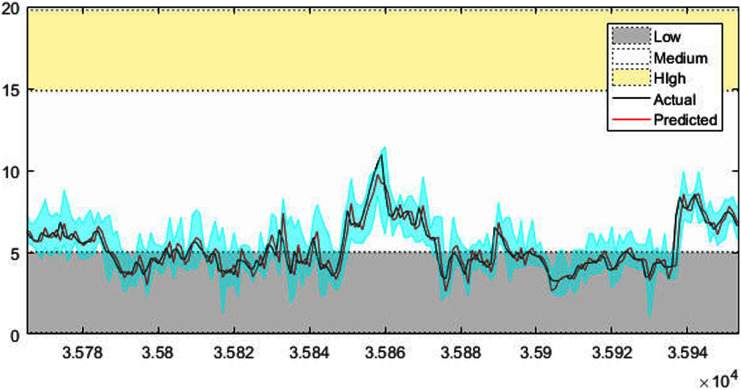
The prediction results of wind speed.

Figures 4–7 shows the test set of these four data sets, the prediction curve, and the prediction interval obtained by using the dynamic weight method. Figures 4–6 shows the prediction of all test sets of the corresponding data sets. Due to a large amount of wind speed data, part of the data are intercepted in Fig. 7 for display. The black curve represents the actual value, the red curve represents the predicted value, and the cyan area represents the predicted interval. The gray area, white and yellow band area in the figure corresponds to the semantics of “low amplitude”, “medium amplitude” and “high amplitude” respectively. Observing these figures, it can be seen that the proposed model can not only obtain the accurate value of the predicted value, but also obtain the interval value of the predicted value, and can describe the predicted value semantically. Thus, the data are described from multiple dimensions, which is more convenient for people to understand.

In order to quantitatively illustrate the performance of the multi-modal prediction model, the experimental results of each data set under the corresponding evaluation index are given in Table 3. By observing the data in the table, it can be found that the method using average weight and model weight has a higher PICP value, which indicates that the probability of prediction interval containing an actual value is higher. However, the PINAW index of the average weight method and the model weight method is higher, that is, the interval distance is relatively wide, which means that the accuracy of the interval is sacrificed to improve the coverage. Although the PICP index of the dynamic weight method is slightly lower, the PINAW index is smaller, which indicates that the prediction interval generated by this method is narrower and the accuracy is higher. The value of the comprehensive index CWC can better reflect the advantages and disadvantages of each method. It can be seen from the table that the dynamic weight method in all the data set experiments has a smaller CWC value. This shows that it is not appropriate to give all sub-modes the same weight or weight does not change. Further, observe the numerical prediction accuracy index RMSE generated by each weighting method in the table, similar conclusions can also be found. The RMSE value generated under the dynamic weight is the smallest, which indicates that the numerical accuracy of the prediction is higher.

**Table 3:** Experimental results.

DataSet	Weight method	PICP	PINAW	CWC	RMSE
MG	dynamic weight	0.9125	0.1472	0.2011	0.0265
	model weight	0.9208	0.2925	0.3998	0.1169
	average weight	0.9500	0.3571	0.4883	0.1074
Exchange rate	dynamic weight	0.8730	0.0959	0.1309	0.0168
	model weight	1.0000	0.6622	0.9058	0.1762
	average weight	1.0000	0.6736	0.9214	0.2031
Vatnsdalsa	dynamic weight	0.8676	0.0685	0.0934	0.7600
	model weight	0.9041	0.1355	0.1852	1.5496
	average weight	0.9087	0.1480	0.2022	1.7385
Wind speed	dynamic weight	0.8701	0.1281	0.1748	0.7626
	model weight	0.9761	0.3141	0.4297	1.2611
	average weight	0.9763	0.3392	0.4639	1.5287

**Figure 8:**
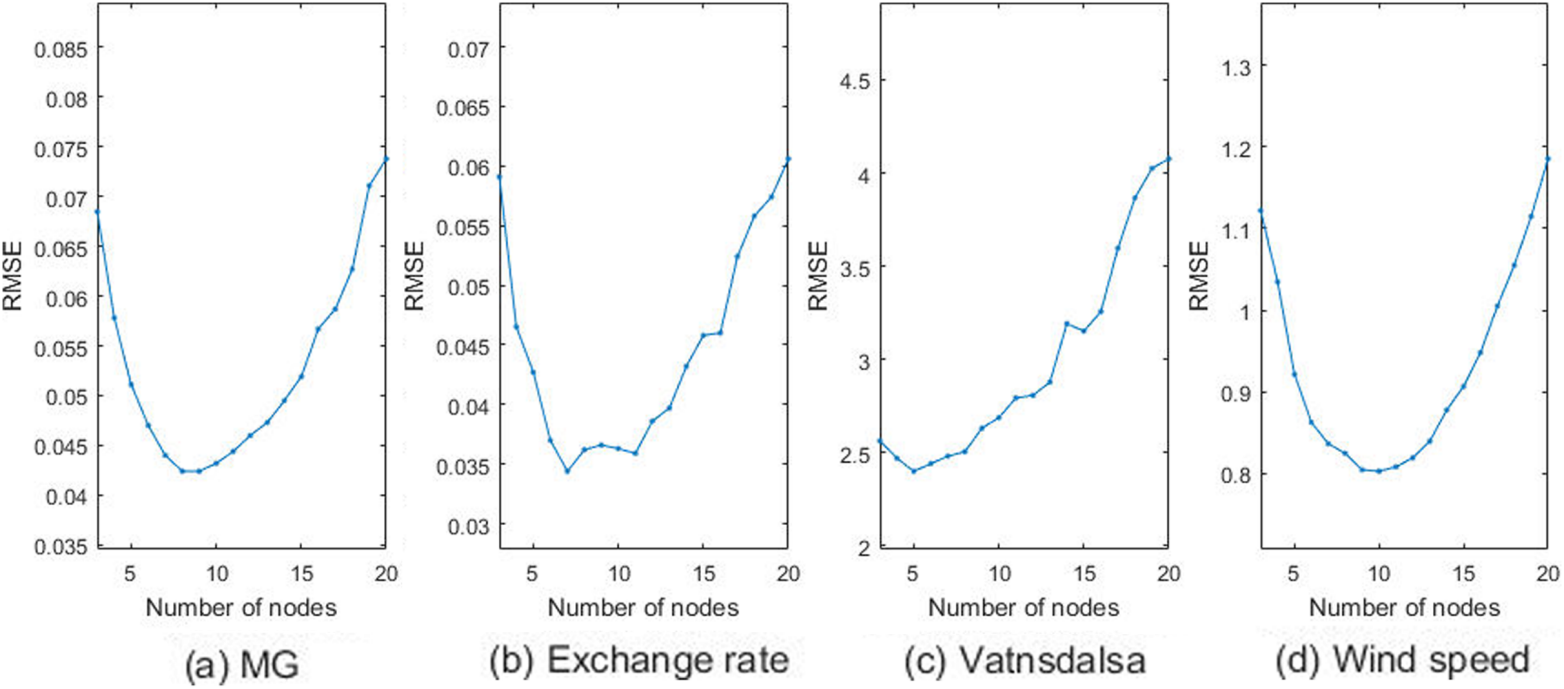
Plots of RMSE *vs* varying number of nodes with overall data. (A) MG, (B) exchange rate, (C) vatnsdalsa, (D) wind speed.

To further analyze the performance of the proposed model, it is compared with the method of training FCMs by using all data. Figure 8 shows the RMSE curve of training FCMs with the whole data, in which the number of traversal nodes ranges from 3 to 20. As can be seen from Fig. 8, the predicted RMSE decreases first with the increase of nodes, but when the number of nodes exceeds a certain value, the RMSE of the predicted value will increase instead. It shows that increasing the number of nodes of FCMs cannot improve the prediction accuracy, and the excessive number of nodes increases the complexity of the model. From Fig. 8, it can be seen that the optimal number of nodes for the four data are 8, 7, 5, and 10 when modeling FCMs with the overall data.

Table 4 shows the RSME comparison results of the proposed method, the single method and LSTM(Long Short-Term Memory)method for time series data prediction accuracy. Among them, the number of nodes selected by the overall modeling method is the best, the LSTM selected has 200 hidden units, and the maximum number of iterations is 250. It can be seen from the table that the multi-modal method has achieved high prediction accuracy.

**Table 4:** Comparison of prediction RMSE between multimodal model and other models.

Data	Muti-modal	Overall model		LSTM
		RMSE	Node number	
MG	0.0265	0.0424	8	0.0348
Exchange rate	0.0168	0.0344	7	0.0225
Vatnsdalsa	0.7600	2.4006	5	1.2041
Wind speed	0.7626	0.8028	10	0.8416

Table 5 shows the operation time of each algorithm on different data. The overall data training model takes the least time. The overall data training model took the least time. The granulation process of the multimodal model was time-consuming, it was necessary to traverse and calculate the sequence data predicted by all sub-models in order to obtain the interval that maximized the Eq. (18). Its time complexity is $n\left( {n - 1} \right)/2$, which means that the time complexity of the algorithm is ${\rm {\rm O}}\left( {{n^2}} \right)$. LSTM needs to train a large model, so the time consumption of it is the most.

**Table 5:** The execution time of algorithms (s).

Data	Proposed	Single model	LSTM
MG	51.95	1.47	179.41
Exchange rate	46.04	1.16	57.44
Vatnsdalsa	51.26	0.92	157.23
Wind speed	335.05	4.06	4,137.44

## Conclusions

Time series often have a variety of change characteristics, that is, the change of the series is multimodal. If each change mode of the time series is modeled separately and the sub-models are fused effectively, the change rules of the time series can be reflected more effectively. A time series processing framework based on a fuzzy cognitive map is proposed in this paper. In this method, a large number of random time segments are selected to extract the possible modal variation characteristics of time series. Fuzzifying the time series and giving the time series semantic description is more conducive to people's cognition and decision-making. The output of each sub model is granulated and integrated, in the process of granulation, the predicted interval of time series can be obtained by maximizing the weight of the sub-model and minimizing the granulation interval. A variety of weight selection methods are studied for the effective fusion of sub-models. The experimental results show that the dynamic weight method can obtain a better prediction interval index and higher prediction accuracy.

In the proposed method, the bootstrap method is used to select a large number of time segments in order to ensure that all the changing modes of time series can be selected. However, this method will inevitably produce redundant time segments, which will reduce the efficiency of the model. Therefore, it is necessary to study more effective methods of time series change modes. For the weights of the sub-models, three basic weight methods are verified in this paper, and the relevant weight selection method can be further studied to improve the performance of the model. In addition, modeling complex system using distributed FCMs is a feasible scheme. A complex system can be decomposed into multiple subsystems. Each subsystem is used to construct one corresponding FCMs model. Then construct a global FCMs model using these sub FCMs models.

## Supplemental Information

10.7717/peerj-cs.726/supp-1 Supplemental Information 1 Code.

10.7717/peerj-cs.726/supp-2 Supplemental Information 2 Exchange rate of Australian dollars.

10.7717/peerj-cs.726/supp-3 Supplemental Information 3 The average daily flow (m3/s) of the Vatnsdalsa River from January 1, 1972 to December 31, 1974, there are 1095 samples, as shown in Figure 3C.

10.7717/peerj-cs.726/supp-4 Supplemental Information 4 The time interval of the data set is 10 minutes. There are 39195 samples as shown in Figure 3D.

## Additional Information and Declarations

### Data Availability

The following information was supplied regarding data availability: Raw data and code are available in the Supplemental Files.
